# Gait speed using powered robotic exoskeletons after spinal cord injury: a systematic review and correlational study

**DOI:** 10.1186/s12984-015-0074-9

**Published:** 2015-10-14

**Authors:** Dennis R. Louie, Janice J. Eng, Tania Lam, Spinal Cord Injury Research Evidence (SCIRE) Research Team

**Affiliations:** University of British Columbia, Vancouver, Canada; Rehabilitation Research Program, 4255 Laurel Street, Vancouver, BC Canada V5Z 2G9; Vancouver Coastal Health Research Institute, Vancouver, Canada; International Collaboration on Repair Discoveries, 818 West 10th Avenue, Vancouver, BC Canada V5Z 1M9; Department of Physical Therapy, University of British Columbia, 212-2177 Wesbrook Mall, Vancouver, BC Canada V6T 1Z3

## Abstract

Powered robotic exoskeletons are an emerging technology of wearable orthoses that can be used as an assistive device to enable non-ambulatory individuals with spinal cord injury (SCI) to walk, or as a rehabilitation tool to improve walking ability in ambulatory individuals with SCI. No studies to date have systematically reviewed the literature on the efficacy of powered exoskeletons on restoring walking function. Our objective was to systematically review the literature to determine the gait speed attained by individuals with SCI when using a powered exoskeleton to walk, factors influencing this speed, and characteristics of studies involving a powered exoskeleton (e.g. inclusion criteria, screening, and training processes). A systematic search in computerized databases was conducted to identify articles that reported on walking outcomes when using a powered exoskeleton. Individual gait speed data from each study was extracted. Pearson correlations were performed between gait speed and 1) age, 2) years post-injury, 3) injury level, and 4) number of training sessions. Fifteen articles met inclusion criteria, 14 of which investigated the powered exoskeleton as an assistive device for non-ambulatory individuals and one which used it as a training intervention for ambulatory individuals with SCI. The mean gait speed attained by non-ambulatory participants (*n* = 84) while wearing a powered exoskeleton was 0.26 m/s, with the majority having a thoracic-level motor-complete injury. Twelve articles reported individual data for the non-ambulatory participants, from which a positive correlation was found between gait speed and 1) age (r = 0.27, 95 % CI 0.02–0.48, *p* = 0.03, 63 participants), 2) injury level (r = 0.27, 95 % CI 0.02–0.48, *p* = 0.03, 63 participants), and 3) training sessions (r = 0.41, 95 % CI 0.16–0.61, *p* = 0.002, 55 participants). In conclusion, powered exoskeletons can provide non-ambulatory individuals with thoracic-level motor-complete SCI the ability to walk at modest speeds. This speed is related to level of injury as well as training time.

## Introduction

The inability to walk is arguably one of the most notable impairments that individuals experience after spinal cord injury (SCI). Besides leading to physical complications such as skin breakdown, muscle atrophy, reduced cardiorespiratory capacity, and pain [1], being unable to walk also affects psychological well-being and can increase the risk of depression and reduce quality of life [2]. For these reasons, recovery of walking consistently ranks among the top priorities related to mobility for individuals with SCI [3]. Unfortunately, a large proportion of these individuals with complete or incomplete injury have limited, if any, recovery of walking function and are thus limited to a wheelchair for their mobility [4]. Even with the use of conventional bracing for ambulation, individuals with SCI must expend high levels of energy [5, 6] to achieve modest, non-functional gait speeds [6, 7], dependent on their level of injury [6].

Recent developments in gait orthoses have produced the powered robotic exoskeleton, a rechargeable bionic device worn over the lower extremities with motorized joints that can provide externally-powered gait independent of a treadmill system [8]. Compared to treadmill-based gait orthoses such as the Lokomat (Hocoma, Switzerland) and LOPES (University of Twente, Netherlands), these powered robotic exoskeletons are compact, lightweight, and portable [9]. This new technology has been designed as an assistive device to provide individuals with complete paralysis the ability to stand and walk independently over-ground in a natural, full weight-bearing, reciprocal pattern. They can also be used in the rehabilitation setting as a training tool to improve stepping and weight-shifting for ambulatory individuals with SCI [9]. Various designs have been developed, several of which are commercially available and are in the process of being approved for use at home and in the community. As with any form of gait rehabilitation, walking with a powered exoskeleton requires specialized training and practice.

As a newly developed technology, the current evidence base surrounding the use of powered robotic exoskeletons in SCI rehabilitation consists of a number of studies, but the majority are case studies (single-subject reports) or single-intervention trials with a small number of participants. A recent systematic review found that energy consumption was reduced when walking with powered orthoses compared to conventional orthoses in paraplegic SCI [10]. A literature review by the same author found that powered gait orthoses have a beneficial effect on the kinematics and temporal-spatial parameters of gait, but reported minimally on gait speed [11]. To our knowledge, no systematic reviews have specifically determined the gait speed attained by non-ambulatory individuals with SCI while using a powered robotic exoskeleton to walk. We defined non-ambulatory individuals with SCI as those who do not walk regularly, independently, with or without gait aids or bracing. Gait speed is an important indicator and will contribute to the utility of the device; very slow speeds may relegate the device to uses solely for exercise, while faster speeds may enable community ambulation.

The primary objective of this article was to examine the evidence on the ability of powered robotic exoskeletons to provide gait, specifically focusing on gait speed, in individuals with SCI by performing a systematic review of relevant clinical studies. To provide continuity across the studies and address the heterogeneity of the presentation of individuals with SCI, we collected individual participant data from each study to explore correlations between participant characteristics and gait speed. We hypothesized that gait speed would be positively correlated with spinal cord preservation (lesion level), as well as training time. Before acquiring a powered robotic exoskeleton, clinicians and users alike should have an understanding of the feasibility of powered exoskeleton use. Thus, secondary objectives were to summarize the (1) screening process for determining suitability for an exoskeleton and the (2) training process to habituate an individual with SCI to walk with an exoskeleton.

## Review

### Methods

We conducted this systematic review according to the PRISMA guidelines and the review protocol is available from the authors [12]. We accessed MEDLINE (1946 to May 6, 2015), EMBASE (1980 to May 6, 2015), Cochrane Central Register of Controlled Trials (1991 to May 6, 2015), and CINAHL (1982 to May 6, 2015). An electronic database search was first conducted using the terms “spinal cord injury” OR “SCI” OR “quadriplegia” OR “tetraplegia” OR “paraplegia” paired using AND with “walk” OR “walking” OR “gait” OR “ambulation”. The search results were then paired using AND with “exoskeleton” OR “exoskeletal” OR “powered gait orthosis” OR “PGO” OR “ReWalk” OR “Ekso” OR “indego” OR “hybrid assistive limb” OR “HAL”. English language and human studies were used as restrictions. Hand searches of reference lists from retrieved articles were completed. Titles, abstracts, and full-texts were screened by two independent reviewers; only studies that met inclusion criteria were selected and used for further analysis.

Eligibility criteria were studies that evaluated walking outcomes of individuals with SCI after training with a powered robotic exoskeleton. We defined powered exoskeleton as a multi-joint orthosis that uses an external power source to move at least two joints on each leg, which is portable, and can be used independent of a treadmill or body-weight support. Papers were selected if they reported gait speed by use of relevant over-ground walking tests (e.g. 10-Meter Walk Test) or temporal-spatial measures relevant to walking (step length, distance, time walking). Additional inclusion criteria were: (1) adult patients over 18 years of age; and (2) peer-reviewed full articles published or “in press”. Exclusion criteria were studies that only utilized a combination of functional electrical stimulation (FES) with the exoskeleton (hybrid exoskeleton), studies that only reported joint angle and muscle moments, or studies that utilized populations with mixed diagnoses (e.g. SCI and stroke) and did not separate the results. Abstracts and conference posters were excluded, as were studies that utilized an orthosis powered only at one joint or a treadmill for testing.

Design characteristics, inclusion and exclusion criteria, sample demographics, exoskeleton characteristics, training protocol, and adverse events were extracted from each study. Individual participant demographics and walking data were extracted from studies, when available, and compiled for statistical analysis. Gait speed was calculated, when not directly reported, from walking outcomes such as the 10-Meter Walk Test (10MWT) or other timed measure. We used the Pearson product-moment correlation coefficient to determine relationships between common participant variables available from the studies (age, years post-injury, injury level, number training sessions) and independent gait speed (hence, without assistance) while walking within the exoskeleton device. We omitted individuals with motor-incomplete injuries from these calculations due to the heterogeneous presentation of incomplete SCI. Injury level was coded as 0 to 17, representing C4 to L1.

## Results

**Fig. 1:**
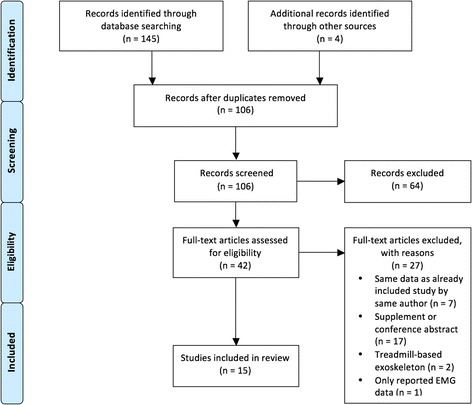
Study results during PRISMA phases: a flowchart of selection process based on inclusion/exclusion criteria

As illustrated in Fig. 1, our search results yielded 145 records across the MEDLINE, CINAHL, EMBASE, and Cochrane databases. After screening for eligibility, 15 articles [13–27] were included in this review (Table 1); seven eligible records were excluded from the final count due to overlapping of participant data. All but two records [14, 24] provided individual participant data that could be extracted for statistical analysis.

**Table 1:** Characteristics of studies included in the review

Authors	Exoskeleton	Use of the exoskeleton	Participants	Walking outcome measures	Training period
Aach et al. (2014) [13]	HAL	Training tool	8 (AIS A to D, T8 to L2)	6MWT, 10MWT, TUG	5d/wk for 90 days, 90 min per session
Arazpour et al. (2013) [14]	Custom powered IRGO	Assistive device	5 (AIS A/B, T6 to T12)	Gait speed, distance	3d/wk for 8 weeks, 2 h per session
Benson et al. (2015) [15]	ReWalk	Assistive device/Training Tool	5 (AIS A/C), C7 to L1	6MWT, 10MWT, TUG	2d/wk for 10 weeks, 2 h per session
Esquenazi et al. (2012) [16]	ReWalk	Assistive device	12 (AIS A/B, T3 to T12)	6MWT, 10MWT	3d/wk for 8 weeks, 75–90 min per session
Evans et al. (2015) [17]	Indego	Assistive device	5 (AIS A, T6 to T12)	6MWT (self-selected pace, fast pace)	At least 5 sessions
Farris et al. (2014) [18]	Indego	Assistive device	1 (AIS A, T10)	6MWT, 10MWT, TUG	20 sessions in one year
Fineberg et al. (2013) [19]	ReWalk	Assistive device	6 (AIS A/B, T1 to T11)	Gait speed	3d/wk for up to 6 months, 1–2 h per session
Hartigan et al. (2015) [20]	Indego	Assistive device	16 (AIS A to C, C5 to L1)	6MWT, 10MWT	5 sessions, 90 min per session
Kolakowsky-Hayner et al. (2013) [21]	Ekso	Assistive device	7 (AIS A, T4 to T12)	Walking distance, time	6d/wk for 1 week, up to 60 min per session
Kozlowski et al. (2015) [22]	Ekso	Assistive Device	7 (AIS A to C, C4 to L1)	2MWT, longest walk	Up to 24 sessions, up to 2 h per session
Kressler et al. (2013) [23]	Ekso	Assistive device	3 (AIS A, T1/2 to T9/10)	Gait speed, distance	3d/wk for 6 weeks, 60 min per session
Neuhaus et al. (2011) [24]	Mina	Assistive device	2 (AIS A, T10 and T12)	Gait speed	9 sessions
Tanabe et al. (2013) [25]	WPAL	Assistive device	7 (AIS A/B, T6 to T12)	Walking distance, time	2–11 sessions, 60 min per session
Yang et al. (2015) [26]	ReWalk	Assistive Device	12 (AIS A to C, C8 to T11)	6MWT, 10MWT	Up to 102 sessions, 1–2 h per session
Zeilig et al. (2012) [27]	ReWalk	Assistive device	6 (AIS A/B, T5 to T12)	6MWT, 10MWT, TUG	Until able to walk 100 m unassisted

*HAL* *Hybrid Assistive Limb*; *6MWT*  Six Minute Walk Test; *10MWT*  Ten Meter Walk Test; *TUG*  Timed Up and Go Test; *IRGO*  Isocentric Reciprocal Gait Orthosis; *2MWT*  Two Meter Walk Test; *WPAL*  Wearable Power-Assist Locomotor

### Study design

The 15 studies ranged from single-subject case studies to prospective intervention trials comparing other types of orthoses within the study. Thirteen studies [14, 16–27] used the powered exoskeleton as an assistive device for ambulation, and thus were post-test studies; in these studies, outcomes were only measured while wearing the device, after a period of training, since individuals did not have the ability to walk without the device (mostly participants with complete injuries). One study [13] used the powered exoskeleton as a training intervention to improve ambulation, assessing walking without the device in a pre-post study design in participants with incomplete and low-complete ambulatory SCI. One study [15] used the powered exoskeleton as both an assistive device as well as training intervention, as they included motor complete and ambulatory incomplete SCI. No studies included a control group and all participants received the powered exoskeleton as their intervention. Two studies [14, 18] compared a powered exoskeleton to standard rigid orthoses, where the same participants trialed multiple orthoses.

### Exoskeletons

Of the 15 studies included, 12 studies [13, 15–23, 26, 27] used commercially developed exoskeletons, such as the ReWalk (ReWalk Robotics, Israel), Ekso (Ekso Bionics, USA), and the Indego (Parker Hannifin Corporation, USA). Two studies investigated exoskeletons developed for research purposes: the Wearable Power Assist Locomotor (WPAL) [25] and Mina [24]. One study [14] utilized a custom device designed by the authors in a previous study [28], an isocentric reciprocating gait orthosis (IRGO) combined with electrically actuated motors. All exoskeletons in the included studies were actuated at the hip and knee joints. The control of walking while wearing a powered exoskeleton varies, with some exoskeletons having multiple control options (Table 2).

**Table 2:** Control options to initiate stepping for powered exoskeletons included in this review

Exoskeleton	External operator	User-operated via buttons	User-operated via weight shifts	User-operated via bioelectric signal detection
ReWalk	•		•	
Ekso	•	•	•	
Indego	•	•	•	
HAL	•			•
Mina	•			
WPAL		•		
Custom IRGO	•			

*HAL* Hybrid Assistive Limb, *WPAL* Wearable Power-Assist Locomotor, *IRGO* Isocentric Reciprocal Gait Orthosis

### Inclusion/exclusion criteria

Ten of the 14 studies using the powered exoskeleton as an assistive device for non-ambulatory individuals with SCI included motor complete or complete SCI (AIS A/B), while three [15, 20, 26] included incomplete SCI (AIS C). One study [22] allowed any participant with lower extremity weakness or paralysis to be eligible, and provided SCI-specific data. Seven studies [15–17, 20, 22, 26, 27] allowed cervical-level injuries to be eligible; the rest of the studies either listed thoracic-level injury or below T1 to qualify for the inclusion criteria. Thirteen studies [13, 15–23, 25–27] specified height and weight restrictions, generally within the range of 1.45 m to 2.0 m and less than 113 kg. Time post-injury for inclusion varied as well, when mentioned, with one study [21] setting a maximum time of two years post-injury, and five studies setting a minimum time post-injury of six months [16, 26, 27] or one year [15, 23]. Three studies [16, 24, 27] required participants to be a regular RGO user in order to be included, and two [15, 22] required participants to be able to maintain an upright posture with or without a standing device.

The study [13] using the powered exoskeleton solely as a training tool for ambulatory individuals included only those with traumatic SCI to the conus medullaris/cauda equina with chronic incomplete or complete paraplegia with extensive zones of partial preservation. Regardless of completeness, they required participants to have some volitional motor function of the hip and knee extensor and flexor groups in order to use the Hybrid Assistive Limb (HAL) exoskeleton (Cyberdyne, Japan), which detects the user’s bioelectrical signals to generate stepping.

Of all the studies included in this review, 11 of the studies had stated exclusion criteria. The exclusion criteria generally consisted of severe comorbidities that would make it unsafe for the participant to use the powered exoskeleton: concurrent neurological or other progressive disease [14–16, 21, 25–27]; unstable spine, fracture risk or osteoporosis [13, 15–17, 21–23, 25–27]; cardiorespiratory limitations to exercise such as autonomic dysreflexia [13, 16, 17, 21–23, 25, 26]; pressure sores at point of contact [13, 16, 17, 21–23, 25–27]; severe limitations in range of motion due to contracture, heterotypic ossification, or spasticity [13–17, 21–23, 25, 26]; or cognitive deficits [13, 15, 16, 21, 25, 27]. Other exclusion criteria were pregnancy [17, 21, 23], asymmetric hip positions [14, 23], surgery in the last three months [23], participation in lower extremity conditioning in last three months [23], previous use of any robotic exoskeletal device [15], Type I or II Diabetes [23], and pain limiting forearm crutch use [23]. Only one study [13] listed non-traumatic SCI as an exclusion criteria for their study.

### Powered exoskeleton as an assistive device for ambulation

#### Participants and level of impairment

There were 92 participants (74 males) across the 14 studies that utilized a powered exoskeleton as an assistive device for ambulation. Of these participants, the majority were motor complete (AIS A or B) thoracic-level SCI (Table 1); six participants had incomplete SCI. The highest level of injury included was C4 and the lowest was L1 with a mean injury level of T7. Participants ranged from two months post-injury to 24 years, with a mean of 5.8 years (SD: 5.6 years) after injury. The mean age of participants across all studies was 37.5 years (SD: 12.3 years).

#### Gait speed

Of the 14 studies utilizing the powered exoskeleton as an assistive device, eight studies [15, 16, 18–20, 23, 26, 27] assessed gait speed by means of the 10MWT, while two studies [14, 24] simply reported gait speed. Two studies [21, 25] reported walking parameters (time and distance) recorded during a session that could be used to calculate a gait speed; these session durations were generally quite long, ranging from 4.5 to 54 min. Two studies [17, 22] calculated gait speed from measures of endurance: the 2-Minute Walk Test (2MWT) and 6-Minute Walk Test (6MWT). Twelve studies reported individual participant gait speed, which ranged ranged from 0.031 m/s to 0.71 m/s. The mean gait speed attained by the 84 participants in these 12 studies was 0.26 m/s (SD: 0.15 m/s) (Table 3).

**Table 3:** Mean gait speed of non-ambulatory participants while using exoskeleton at end of training period

				Gait speed (m/s) Mean (SD)
Participants with individual data (*n* = 84)				0.26 (0.15)
	Incomplete SCI participants (*n* = 6)			0.32 (0.25)
	Complete SCI participants (*n* = 78)			0.25 (0.14)
		By device		
			ReWalk (*n* = 37)	0.28 (0.15)
			Ekso (*n* = 18)	0.14 (0.07)
			Indego (*n* = 20)	0.31 (0.11)
			WPAL (*n* = 7)	0.16 (0.06)
		By assistance^a^		
			No hands-on assistance (*n* = 63)	0.26 (0.15)
			Hands-on assistance (*n* = 15)	0.21 (0.07)

*SD* Standard Deviation, *WPAL* Wearable Power-Assist Locomotor

^a^Hands-on physical assistance provided during evaluation of gait speed

#### Gait aid at assessment

At this time, powered exoskeletons require the use of a gait aid for support during stepping. The general expectation is for exoskeleton users to eventually progress to forearm crutches, which provide less stability than walking frames but are less bulky and thus more portable. One study [20] which included individuals with cervical-level SCI, allowed participants to use a platform walker if needed. Seven studies [14, 17, 18, 20, 21, 23, 26] allowed participants to use a 2-wheeled walker for assessment; 10 studies [15–17, 19–22, 24, 26, 27] had participants who achieved exoskeletal walking with forearm crutches by the end of the training period.

#### Control of exoskeleton and independence

The control of walking while wearing a powered exoskeleton varies (Table 2). In two studies [23, 25], participants ambulated by controlling stepping with buttons on their walker. In 10 studies [15–20, 22, 23, 26, 27], participants generated stepping by shifting their own weight within the exoskeleton; the exoskeleton is able to detect changes in centre of mass over one limb and in response generates a step contralaterally. In another three studies [14, 21, 24], exoskeletal stepping was initiated by an external operator using a control interface. While all studies reported on participants that did not require assistance, four studies [19, 20, 22, 26] also reported on several participants requiring minimal to moderate hands-on assistance with the exoskeleton during the gait assessment. The mean gait speeds attained by participants using the exoskeleton as an assistive device, grouped by exoskeleton, level of assistance, and completeness of injury, are shown in Table 3.

#### Training protocol

As seen in Table 1, training period varied significantly across the studies included in this review. Some studies involved a shorter training period [17, 20, 21, 24, 25, 27], often ending when the exoskeleton user achieved independence or the ability to walk a set distance; other studies [14–16, 19, 22, 23] utilized a set training protocol lasting several weeks to months, not based on participant progress. One study [26] did not have a set training protocol or end-point, with participants undergoing between 12 and 102 training sessions to achieve their best performance with the exoskeleton. An aggregate mean of 19.8 (SD = 18.6, *n* = 79) training sessions was calculated across all studies; training sessions were 60 to 120 min in duration. In all studies, participants were generally progressed from standing in the exoskeleton to weight shifting and stepping exercises to walking either within parallel bars or using a gait aid. In three studies [15, 19, 22], participants were progressed to training on different surfaces including sidewalk, grass, or stairs. Tanabe et al. [25] incorporated a treadmill as part of the training protocol to improve user confidence and speed. Only one study included upper extremity strengthening and lower extremity stretching as part of the intervention protocol [14].

#### Powered exoskeleton as a training tool to improve ambulation

As an intervention for ambulatory individuals with SCI, eight participants trained with the HAL in the Aach et al. [13] study for five days a week over a 90-day period (mean of 51.75 sessions). Participants ambulated on a body weight-supported treadmill while wearing the HAL; speed and body weight-support were adjusted individually. At the end of the intervention period, the participants improved their mean gait speed without the exoskeleton from 0.28 m/s to 0.50 m/s (*p* < 0.05, *n* = 8, effect size = 0.71). They also demonstrated an improvement in mean 6MWT distance from 70.1 m to 163.3 m (*p* < 0.05, *n* = 8, effect size = 0.64). On the other hand, the two participants with incomplete SCI in the Benson et al. [15] study did not show clear improvements in mean gait speed (0.26 m/s to 0.27 m/s) or 6MWT distance. In contrast to the Aach et al. [13] study, these two participants underwent only 20 training sessions over 10 weeks, which did not include continuous treadmill training.

#### Adverse events

Across all 15 articles, five [14, 17–19, 25] did not report on whether any adverse events occurred with use of a powered exoskeleton. Of the 10 studies that reported on adverse events, five [13, 22–24, 27] reported no skin changes, while five [15, 16, 20, 21, 26] reported mild skin effects (redness or superficial abrasions). Four articles [16, 21, 23, 24] addressed and reported no change in spasticity, and five [16, 21, 23, 24, 27] which addressed pain reported no change or a slight decrease in usual pain. Safety precautions, such as overhead tether or close guarding, were taken in all studies to ensure participant safety, though loss of balance was used for some articles as an outcome measure. In one study [21], falls engaging the overhead tether were reported for three participants over six days of training; two of these participants experienced a combined three falls due to mechanical programming errors of the exoskeleton, while the third participant experienced over 10 falls due to malfunctioning of specialized forearm crutches which were later discontinued. Two studies [16, 24] reported some lower extremity edema due to prolonged standing. One study [15] removed a participant for safety reasons due to a “near-serious” device-related adverse event involving a hairline fracture of the talus that did not require treatment.

#### Factors influencing exoskeletal gait speed in non-ambulatory individuals with SCI

Four variables were found in the majority of studies which might influence gait speed in non-ambulatory individuals using the exoskeleton device to walk: age, injury duration, injury level, and number of training sessions. As the number of incomplete participants across all the studies was small (*n* = 6), they were not included in the correlational analyses. We also removed the participants who required hands-on assistance to ambulate with the exoskeleton from the correlation calculations.

**Fig. 2:**
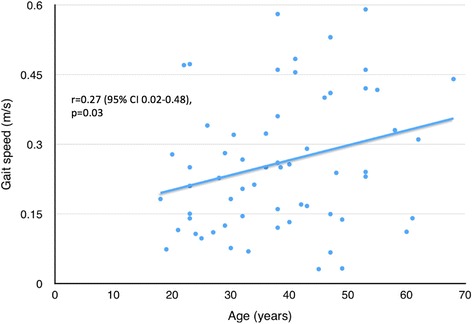
Gait speed plotted against age using individual participant data, excluding those with incomplete injuries or requiring assistance to ambulate (*n* = 63 from 12 studies)

**Fig. 3:**
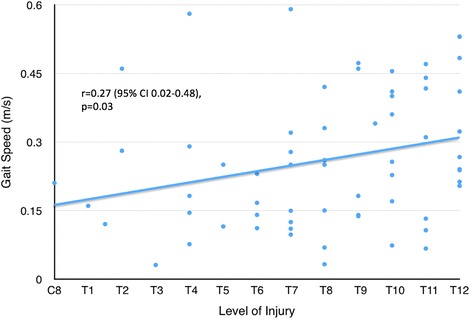
Gait speed plotted against injury level using individual participant data, excluding those with incomplete injuries or requiring assistance to ambulate (*n* = 63 from 12 studies)

All 12 studies reporting individual data provided information on participant age; in some cases, a narrow age range (e.g. 20–24) was provided, and the midpoint of the range was used for that individual. A significant correlation was found between increasing age and faster gait speed (r = 0.27, 95 % CI 0.02–0.48, *p* = 0.03, *n* = 63) (Fig. 2). However, no relationship was found between injury duration and gait speed (r = 0.19, 95 % CI −0.09–0.44, *p* = 0.18, *n* = 53) from 10 studies. From the 12 studies, we found a significant correlation between injury level and gait speed (r = 0.27, 95 % CI 0.02–0.48, *p* = 0.03, *n* = 63). Higher speeds were associated with a lower level of injury when walking with an exoskeleton as an assistive device (Fig. 3).

**Fig. 4:**
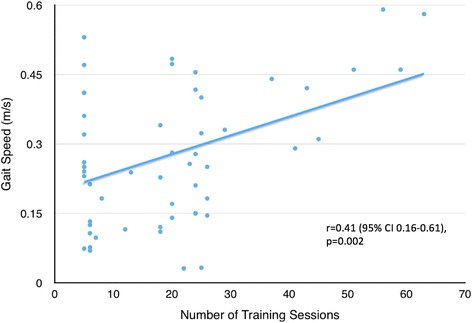
Gait speed plotted against number of training sessions using individual participant data, excluding those with incomplete injuries or requiring assistance to ambulate (*n* = 55 from 11 studies, one outlier removed)

Eleven studies reported the number of training sessions for individual participants. Those who were able to practice longer with the powered exoskeleton achieved faster gait speeds (r = 0.27, 95 % CI 0.003–0.49, *p* = 0.048, *n* = 56). One individual in the Yang et al. [26] study underwent 102 training sessions to achieve their best walking outcome, compared to the group mean of 19.8 sessions. When we removed this outlier, the correlation coefficient increased to 0.41 (95 % CI 0.16–0.61; *p* = 0.002, *n* = 55) (Fig. 4).

## Discussion

The advent of the powered exoskeleton in rehabilitation has many implications for individuals with SCI with limited or no walking ability. It allows wheelchair-users to stand and ambulate, which may influence community mobility and social participation. Powered exoskeletons also require less energy to use than standard rigid orthoses [10] and are becoming lighter and more accessible. Use of powered exoskeletons without overhead bodyweight support for over-ground ambulation is a new rehabilitation strategy, and to our knowledge our review is the first to examine their ability for promoting gait speed for individuals with SCI.

The relationship between level of injury and gait speed suggests that proficiency of powered exoskeletal walking is linked to the functional presentation of the user. Individuals with more neurological preservation of their spinal cord are more likely to achieve greater speeds with a powered exoskeleton. Though the upper extremities are considered spared in all thoracic-level SCI, individuals with high thoracic injuries include pectoralis major and latissimus dorsi in their postural control muscle synergies [29]; individuals with greater preservation have more trunk musculature activation and can control their centre of mass with less dependence on the arms. Currently, all powered exoskeletons require the use of an additional gait aid, and some generate stepping in response to lateral shifts of centre of mass. An individual with less reliance on the upper extremities for maintaining postural stability will be more able to lift or push their gait aid and to navigate their centre of mass.

There was an unexpected relationship found between age and gait speed, with older participants achieving greater speeds than younger participants. One possible explanation for this relationship may lie in the epidemiology of SCI. Younger individuals with SCI tend to sustain a traumatic SCI, while older individuals with SCI tend to have a non-traumatic SCI [30, 31]. Further to this, traumatic SCI tends to result in a higher level of injury and more neurological impairment than non-traumatic SCI [32, 33]. Many of the studies included in this review did not indicate whether participants had a traumatic or non-traumatic injury, so we could not confirm this hypothesis. However, a post-hoc analysis found a non-significant trend between increasing age and lower levels of injury (i.e. less neurological impairment) (r = 0.20, 95 % CI −0.05–0.43, *p* = 0.11, *n* = 63). Without controlling for injury level, we would then expect the older individuals in our included studies to walk faster than younger individuals.

Participants were able to ambulate independently within a reasonable training time, with some subjects doing so within the first training session. However, those who were able to train for several weeks to months were generally able to achieve ambulation at faster speeds with a powered exoskeleton. Repetitive task practice is a requirement for improved speed and accuracy of a new skill [34], and is a possible explanation for this relationship. As exoskeletons are beginning to be approved for personal and home use, daily use may help exoskeleton-users attain higher gait speeds quickly.

Our findings showed that use of a powered exoskeleton allowed non-ambulatory individuals with SCI to ambulate at a mean speed of 0.26 m/s, despite the maximum speed of commercial powered exoskeletons such as the ReWalk being 0.55 m/s (ReWalk™ Personal System User Guide, ReWalk Robotics, Israel). A gait speed of 0.26 m/s is not considered sufficient for community ambulation; Forrest et al. [35] found a threshold of 0.44 m/s for limited community ambulation after incomplete SCI while Andrews et al. [36] determined the mean speed necessary to cross an intersection as set by traffic signals to be 0.49 m/s. However, 0.26 m/s is within a range comparable to individuals with incomplete SCI who are able to walk with or without supervision indoors [37]. In our included studies, one individual with a motor-incomplete C8 SCI using a ReWalk was able to ambulate at 0.71 m/s, higher than the device’s reported maximum speed of 0.55 m/s.

As a training intervention for ambulatory individuals with SCI, participants in the Aach et al. [13] study demonstrated significant improvements in gait speed and endurance with use of a powered exoskeleton. These large improvements may be due in part to the principles of motor learning and neuroplasticity. The high level of repetition on the treadmill and feedback of successful active stepping with combined use of the bioelectric signal-dependent HAL exoskeleton may have helped to strengthen the intact neural pathways in incomplete SCI [38]. On the other hand, the two participants in the Benson et al. [15] study with incomplete SCI did not show any improvement in gait speed or endurance. Similarly, a systematic review of treadmill-based robotics-assisted locomotor training found reduced walking endurance and no difference in gait speed after robotics-assisted locomotor training using the Lokomat (Hocoma, Switzerland) compared to other forms of gait training [39]. Due to these mixed findings, further research in this population is required to investigate the potential of powered exoskeletons as a training tool.

The training protocol was similar across all the studies, progressing from becoming familiar with standing and balancing in the exoskeleton to stepping and walking within the exoskeleton. This progression of confidence is similar to training with other lower limb orthoses, with repetition being a key principle for training. All studies employed safety precautions (spotting and overhead tether) to ensure safety and confidence while learning to use a new assistive device.

This systematic review has some limitations. The level of evidence in the current literature is limited to studies with a small number of participants. In addition, a true control group (without a device to walk) is not relevant as most participants would not have been able to walk without the exoskeleton; however, future studies could compare different orthotic, FES, or exoskeleton systems. There was heterogeneity in the study characteristics (device, control of stepping, training duration, outcome measurement), which made it challenging to compare results and reduces the ability to generalize results. However, we attempted to overcome this by aggregating participant data to allow statistical analysis to explore correlations between participant characteristics and outcomes. In the future, it would be useful for studies to report on the exact intensity of training, using such measures as number of steps or walking time.

## Conclusion

In conclusion, powered exoskeletons can provide individuals with thoracic-level motor-complete SCI the ability to walk at modest speeds. Exoskeletal gait speed is related to the amount of time spent practicing as well as level of injury.
